# Supplementary material for: Identification of Freezing-Responsive microRNAs and Their Targets in Chinese Jujube by Small RNA and Degradome Sequencing
Source: Int J Mol Sci. 2026 Sep 4;27(17):7912. doi: 10.3390/ijms27177912 (PMC13566890; doi:10.3390/ijms27177912)
Supplement: Supplementary file 1 [file ijms-27-07912-s001.zip › Supplementary Material S2/novel_miR_90.pdf]

Provisional ID	:	novel_miR_90
Score total	:	0.7
Score for star read(s)	:	-1.3
Score for read counts	:	0
Score for mfe	:	0.4
Score for randfold	:	1.6
Score for cons. seed	:	
Total read count	:	387
Mature read count	:	387
Loop read count	:	0
Star read count	:	0

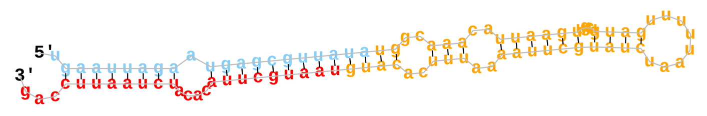

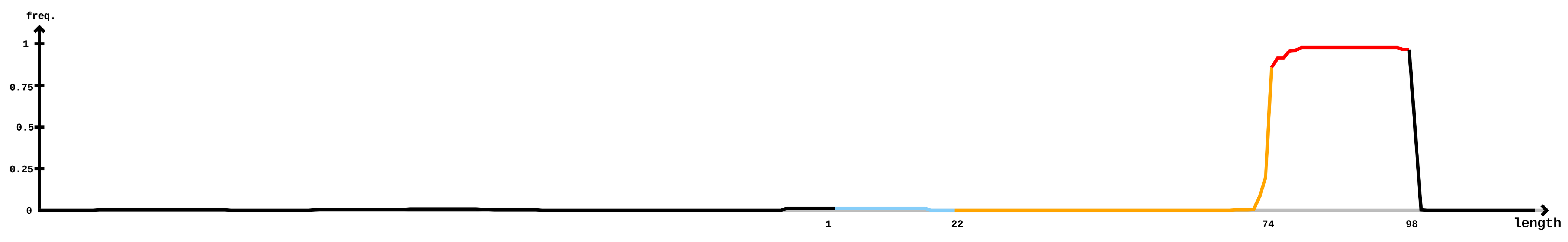

Star Mature

```
5'- gguugcuacaauuuugucucccagcucuggcaugcugcccuuguguugagcauguugauuguaguaguuauggucguccacaugccaugauaugucauuuaugccauugaguacgauuaguuuauugauguugaauuagaaugagcguuauauggcaaacauuaagguugcuguaguuuuuaauucuaugcuuaaaauuucacauguaaugcuuacacaucuaauuccaggauggcaucuguaauaucaaag -3'   exp
    ..((((((((((.((((((...((...((((...))))...))...))).))).)))))))))))...(((.(((....))))))((((((..((...(((((((.....((((......)))).....((((((((.((((((((((((..(((..((((((....((((........))))))))))..))).))))))))))))))....))))))))...)))))))).))..))))))...   reads   mm   sample
    ........caauuuugucucccagcucugg..............................................................................................................................................................................................................................   1   0   LT-3
    ............................................guugagcauguugauuguaguaguuaug....................................................................................................................................................................................   1   0   LT-2
    .............................................uugagcauguugauuguaguaguuauggu..................................................................................................................................................................................   1   0   LT-3
    ............................................................guaguaguuauggucguccaca..........................................................................................................................................................................   1   0   LT-1
    ...........................................................................................................................auugauguugaauuagaaugagcg.........................................................................................................   2   0   JCK-2
    ...........................................................................................................................auugauguugaauuagaaugagcg.........................................................................................................   3   0   JCK-3
    ........................................................................................................................................................................................................cacauguaaugcuuacacaucuaauuccag......................   1   0   JCK-1
    ...........................................................................................................................................................................................................Cuguaaugcuuacacaucuaauuccag......................   1   1   LCK-3
    ............................................................................................................................................................................................................uguaaugcuuacacaucuaauuccag......................   2   0   LT-3
    ............................................................................................................................................................................................................uguaaugcuuacacaucuaauuccag......................   2   0   JCK-2
    ............................................................................................................................................................................................................uguaaugcuuacacaucuaauuccag......................   18   0   LCK-3
    ............................................................................................................................................................................................................uguaaugcuuacacaucuaauuccag......................   2   0   LT-2
    ............................................................................................................................................................................................................uguaaugcuuacacaucuaauuccag......................   1   0   JCK-3
    ............................................................................................................................................................................................................uguaaugcuuacacaucuaauuccag......................   1   0   JCK-1
    ............................................................................................................................................................................................................uguaaugcuuacacaucuaauuccag......................   1   0   LT-1
    ............................................................................................................................................................................................................uguaaugcuuacacaucuaauuccagA.....................   4   1   LT-2
    .............................................................................................................................................................................................................guaaugcuuacacaucuaauNccag......................   1   1   JCK-1
    .............................................................................................................................................................................................................guaaugcuuacacaucuaauuccag......................   3   0   LT-3
    .............................................................................................................................................................................................................guaaugcuuacacaucuaauuccag......................   7   0   LT-1
    .............................................................................................................................................................................................................guaaugcuuacacaucuaauuccag......................   3   0   JCK-1
    .............................................................................................................................................................................................................guaaugcuuacacaucuaauuccag......................   11   0   JCK-2
    .............................................................................................................................................................................................................guaaugcuuacacaucuaauuccag......................   3   0   LT-2
    .............................................................................................................................................................................................................guaaugcuuacacaucuaauuccag......................   14   0   LCK-3
    .............................................................................................................................................................................................................guaaugcuuacacaucuaauuccag......................   3   0   JCK-3
    .............................................................................................................................................................................................................guaaugcuuacacaucuaauuccagga....................   1   0   LT-3
    ..............................................................................................................................................................................................................uaaugcuuacacaucuaauuccag......................   55   0   LCK-3
    ..............................................................................................................................................................................................................uaaugcuuacacaucuaauuccag......................   40   0   JT-1
    ..............................................................................................................................................................................................................uaaugcuuacacaucuaauuccag......................   23   0   LT-1
    ..............................................................................................................................................................................................................uaaugcuuacCcaucuaauuccag......................   1   1   LT-1
    ..............................................................................................................................................................................................................uaaugcuuacacaucuaauuccag......................   1   0   JT-3
    ..............................................................................................................................................................................................................uaaugcuuacacaucuaauuccGg......................   1   1   JCK-1
    ..............................................................................................................................................................................................................uaaugcuuacacaGcuaauuccag......................   1   1   JT-1
    ..............................................................................................................................................................................................................uaaugcuuacacaucuaauuccag......................   20   0   LT-3
    ..............................................................................................................................................................................................................uaCugcuuacacaucuaauuccag......................   1   1   LT-2
```

```
                                                                                                                                    Star                                                                                 Mature
gguugcuacaauuuugucucccagcucuggcaugcugcccuuguguugagcauguugauuguaguaguuauggucguccacaugccaugauaugucauuuaugccauugaguacgauuaguuuauuugauguugaauuagaaugagcguuauauggcaaacauuaaguugcuguaguuuuuaaucuaugcuuaaaaauuucacauguaaugcuuacacaucuaauuccaggauggcaucuguaauaucaaag
..............................................................................................................................................................................................................uaaugcuuacacaucuaauuccag......................   35   0   LT-2
..............................................................................................................................................................................................................uaaugcuuacacaucuaauuccag......................   1    0   JT-2
..............................................................................................................................................................................................................uaaugcuuacacaucuaauuccag......................   16   0   JCK-3
..............................................................................................................................................................................................................uaaugcuuacacaucuaauuccGg......................   1    1   JCK-2
..............................................................................................................................................................................................................uaaugcuuacacaucuaauuccag......................   35   0   JCK-2
..............................................................................................................................................................................................................uaaugcCuacacaucuaauuccag......................   1    1   JT-1
..............................................................................................................................................................................................................uaaugcuuacacaucuaCuuccag......................   1    1   LCK-3
..............................................................................................................................................................................................................uaaugcuuacacaucuaauuccag......................   23   0   JCK-1
..............................................................................................................................................................................................................uaaugcuuacacaucuaauuccagA.....................   1    1   LT-1
..............................................................................................................................................................................................................uaaugcuuacacaucuaauuccagA.....................   5    1   LCK-3
...............................................................................................................................................................................................................aaugcuuacacaucuaauucc........................   3    0   JCK-1
...............................................................................................................................................................................................................aaugcuuacacaucuaauucc........................   2    0   JCK-3
...............................................................................................................................................................................................................aaugcuuacacaucuaauuccag......................   4    0   LT-1
...............................................................................................................................................................................................................aaugcuuacacaucuaauuccag......................   3    0   LT-2
...............................................................................................................................................................................................................aaugcuuacacaCcuaauuccag......................   1    1   LCK-3
...............................................................................................................................................................................................................aaugcuuacacaucuaauuccag......................   5    0   LCK-3
...............................................................................................................................................................................................................aaugcuuacacaucuaauuccag......................   2    0   JCK-2
...............................................................................................................................................................................................................aaugcuuacacaucuaauuccag......................   3    0   LT-3
.................................................................................................................................................................................................................ugcuuacacaucuaauuccag......................   3    0   JT-3
.................................................................................................................................................................................................................ugcuuacacaucuaauuccag......................   6    0   JT-2
.................................................................................................................................................................................................................ugcuuacacaucuaauuccag......................   2    0   LT-1
.................................................................................................................................................................................................................ugcuuacaGaucuaauuccag......................   1    1   LT-2
.................................................................................................................................................................................................................ugcuuacacaucuaauuccag......................   4    0   LT-2
.................................................................................................................................................................................................................ugcuuacacaucuaauuccagA.....................   1    1   LT-2
..................................................................................................................................................................................................................gcuuacacaucuaauuccag......................   1    0   LT-1
...................................................................................................................................................................................................................cuuacacaucuaauuccag......................   1    0   JCK-3
...................................................................................................................................................................................................................cuuacacaucuaauuccag......................   6    0   LCK-3
```
